# Bird Diversity and Habitat Associations in the Tara Gedam Monastery Church Forest: A Seasonally Informed Study in Northwestern Ethiopia

**DOI:** 10.1002/ece3.73789

**Published:** 2026-06-09

**Authors:** Shimelis Aynalem Zelelew, Melkeneh Wondie, Mezgebu Ashagire

**Affiliations:** ^1^ Department of Wildlife and Ecotourism Management College of Agriculture and Environmental Sciences, Bahir Dar University Ethiopia; ^2^ Department of Animal Science, Fisheries, Wetlands and Wildlife Management Program College of Agricultural Science, Arba Minch University Ethiopia

**Keywords:** avian community, edge effect, foraging guilds, habitat structure, sacred grove, seasonal variation, species diversity

## Abstract

The expansion of seasonal agriculture has contributed to forest habitat loss and declines in avian populations dependent on these ecosystems. This study investigates how habitat structure and seasonal variation influence bird species richness, abundance, and community composition in the Tara Gedam Monastery church forest, a sacred grove. The forest was stratified into edge, intermediate, and interior zones based on distance from the edge and vegetation structure. Data were collected during the wet (August–October 2019) and dry (December 2019–February 2020) seasons using 200 point counts across 20 sample blocks. Tree density and diameter at breast height (DBH) were measured within 20 m × 20 m quadrats. Bird community structure was assessed using Shannon diversity, evenness, and equitability indices, alongside Chao‐1 and ACE estimators. A total of 94 species and 2375 individual birds were recorded, with an overall Shannon Diversity Index of 4.009, indicating high species diversity. While bird abundance was 61% higher in the wet season, species richness and diversity remained remarkably stable between seasons, reflecting the forest's role as a perennial refuge. Multivariate analysis revealed that avian community structure is shaped by complex interactions among habitat class, distance from edge, tree density, and tree DBH (Roy's largest root = 1.731, *p* = 0.009). Contrary to classic edge effect predictions, both species richness and abundance were significantly higher at the forest edge, highlighting the ecological importance of ecotone habitats. Foraging guild analysis revealed a community dominated by insectivores (25 species) and omnivores (38 species), with near‐equal representation of arboreal (39 species) and ground‐foraging (41 species) strata, underscoring the importance of vertical habitat heterogeneity. These findings emphasize that the conservation value of sacred groves extends beyond species richness to encompass functional diversity and community resilience. Preserving structural complexity, particularly at forest edges, is critical for sustaining avian biodiversity in fragmented agricultural landscapes.

## Graphical abstract

This study explores how habitat structure and seasonal variation shape bird diversity in the Tara Gedam Monastery church forest of Ethiopia. Across wet and dry seasons, 94 species were recorded, with abundance higher in the wet season but diversity remaining stable, underscoring the forest's role as a perennial refuge. Results highlight the ecological importance of forest edges and structural complexity in sustaining avian biodiversity within fragmented agricultural landscapes.

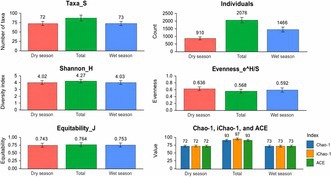

## 1 Introduction

Assessing bird communities is a critical tool in biodiversity conservation, providing a foundation for identifying and prioritizing conservation actions (Sethy et al. 2015). Knowledge of avian diversity and composition is essential for determining the health of local ecosystems and regional landscapes. Species richness and functional diversity profoundly influence ecosystem resilience, providing insurance against environmental change. Consequently, there is growing scientific interest in quantifying functional diversity and understanding how it varies along environmental gradients (Mace et al. 2014).

Forest birds are particularly sensitive to changes in vegetation structure and forest extent due to their reliance on complex vertical stratification and specialized social structures (Martin and Possingham 2005; Davies and Asner 2014). Key habitat features such as floristic complexity, vegetation cover, and density are critical factors in avian habitat selection. These correlated features provide essential resources, food, nesting material, and cover, thereby supporting bird populations. Habitat heterogeneity plays a significant role in determining species abundance and occurrence (Pennington and Blair 2011).

Tropical ecosystems are characterized by pronounced seasonality, with alternating wet and dry periods that strongly influence avian community dynamics (Loiselle and Blake 1991; Poulin et al. 1992). While many studies have focused on fragmented or isolated forests (Mequanint et al. 2024; Zelelew et al. 2025), few have examined seasonal variation within a single, continuous forest system. Investigating these dynamics in the intact Tara Gedam Monastery Church forest is therefore essential for understanding the resilience of undisturbed habitats and their conservation value.

The removal or reduction of vegetation reduces contiguous habitat area, leading to fragmentation and increased isolation, and exposing bird populations to heightened predation risk (Schlossberg and King 2008). Horizontal stratification, expressed through edge, intermediate, and interior forest zones, also significantly structures bird communities. Edges often attract generalist and edge‐dependent species (Chen et al. 1999; Rodewald et al. 2001), while the forest interior, characterized by closed canopies and mature trees, supports specialized, obligate forest birds (Ries et al. 2004). Understanding this horizontal structure is particularly important in Ethiopia's montane forests, where ecological studies remain limited.

Birds provide crucial regulating and supporting ecosystem services through their foraging ecology, including scavenging, nutrient cycling, seed dispersal, pollination, and pest control (Sekercioglu 2006; Whelan et al. 2008). As bioindicators of ecosystem health, their protection is integral to managing biological threats and conserving overall biodiversity. Insectivorous birds and raptors help regulate disease vectors, while scavengers contribute to biomass recycling. Frugivorous birds are key agents of seed dispersal for fleshy‐fruited plants, and nectarivores such as sunbirds play an important role in pollinating specific flowering plants (Stevenson and Fanshawe 2020; Judd 2008).

In Ethiopia, centuries of population pressure have reduced forests to fragmented patches, many of which persist due to religious protection. Forest cover in the northern highlands has declined markedly; for example, woody vegetation in the Lake Tana watershed fell from ~20% in the mid‐20th century to ~10% by 2014–2016 (Frankl et al. 2019). Tara Gedam is one such remnant Afromontane evergreen forest in northwestern Ethiopia, representing a critical biodiversity refuge in a heavily altered landscape (Tessfa et al. 2020).

Ethiopian church forests, including Tara Gedam, are unique socio‐ecological systems where religious reverence has historically preserved native vegetation in otherwise deforested landscapes (Wassie et al. 2005; Cardinale et al. 2017). These sacred groves often serve as biodiversity hotspots, yet the specific relationships between avian diversity and key forest structural variables—such as tree density, diameter at breast height (DBH), and distance from forest edge—remain poorly understood in these habitats. Furthermore, how different foraging guilds (e.g., insectivores, frugivores, nectarivores, granivores) respond to these same forest variables has received limited attention, despite its importance for understanding resource partitioning and community assembly (Kissling et al. 2007; Dehling et al. 2014). Addressing these knowledge gaps is critical because guild‐specific responses can reveal the underlying mechanisms driving avian community structure. For instance, insectivores may depend on high tree density and complex understory cover, whereas frugivores may be more influenced by the presence of specific fruit‐bearing tree species and DBH class distributions (Bibby et al. 1992; Sekercioglu 2006). Understanding these relationships within the intact, large forest of Tara Gedam Monastery provides a valuable baseline against which fragmented church forests can be compared.

Despite its ecological importance, the avifauna of Tara Gedam remains poorly studied, particularly regarding seasonal dynamics and the influence of vegetation structure. To address this gap, this study was designed with three objectives: (1) to evaluate the effect of seasonality on avian diversity; (2) to assess the relationships between vegetation structure (e.g., tree density, diameter at breast height (DBH)) and avian community metrics (richness, evenness, and abundance); and (3) to characterize the functional foraging guilds within the forest ecosystem.

Based on established ecological principles, we formulated the following hypotheses: (i) General bird diversity hypothesis: It was hypothesized that the Tara Gedam Monastery church forest, as an isolated sacred grove, would support a diverse avian assemblage characterized by high species richness and an even distribution of individuals. This prediction is supported that sacred groves have long been recognized for their critical role in biodiversity conservation due to religious and cultural practices that protect flora and fauna from disturbance (Kumar and Koli 2024, 2025; Areaya et al. 2013) (ii) Seasonal variation hypothesis: We hypothesized that avian community structure would vary significantly between the wet and dry seasons in response to seasonal changes in resource availability. This prediction is grounded in the seasonal pulse of resource availability, specifically increased insect and fruit abundance, which typically supports a more diverse and abundant avian assemblage (Levey 1988). (iii) Edge effect and vegetation structure hypothesis: It was hypothesized that the transition from forest edge to interior, along with associated vegetation characteristics, would significantly influence avian community metrics.

This expectation is supported by evidence that vegetation diversity and structural complexity are key positive predictors of bird species richness, as they provide a wider array of niches and resources (Tews et al. 2004; Báldi 2008; Suarez‐Rubio and Thomlinson 2009; Zelelew et al. 2025). Conversely, we expect a negative correlation with distance from the core forest habitat, consistent with the ecological principle of distance decay.

## 2 Methods

### 2.1 Study Area

**FIGURE 1:**
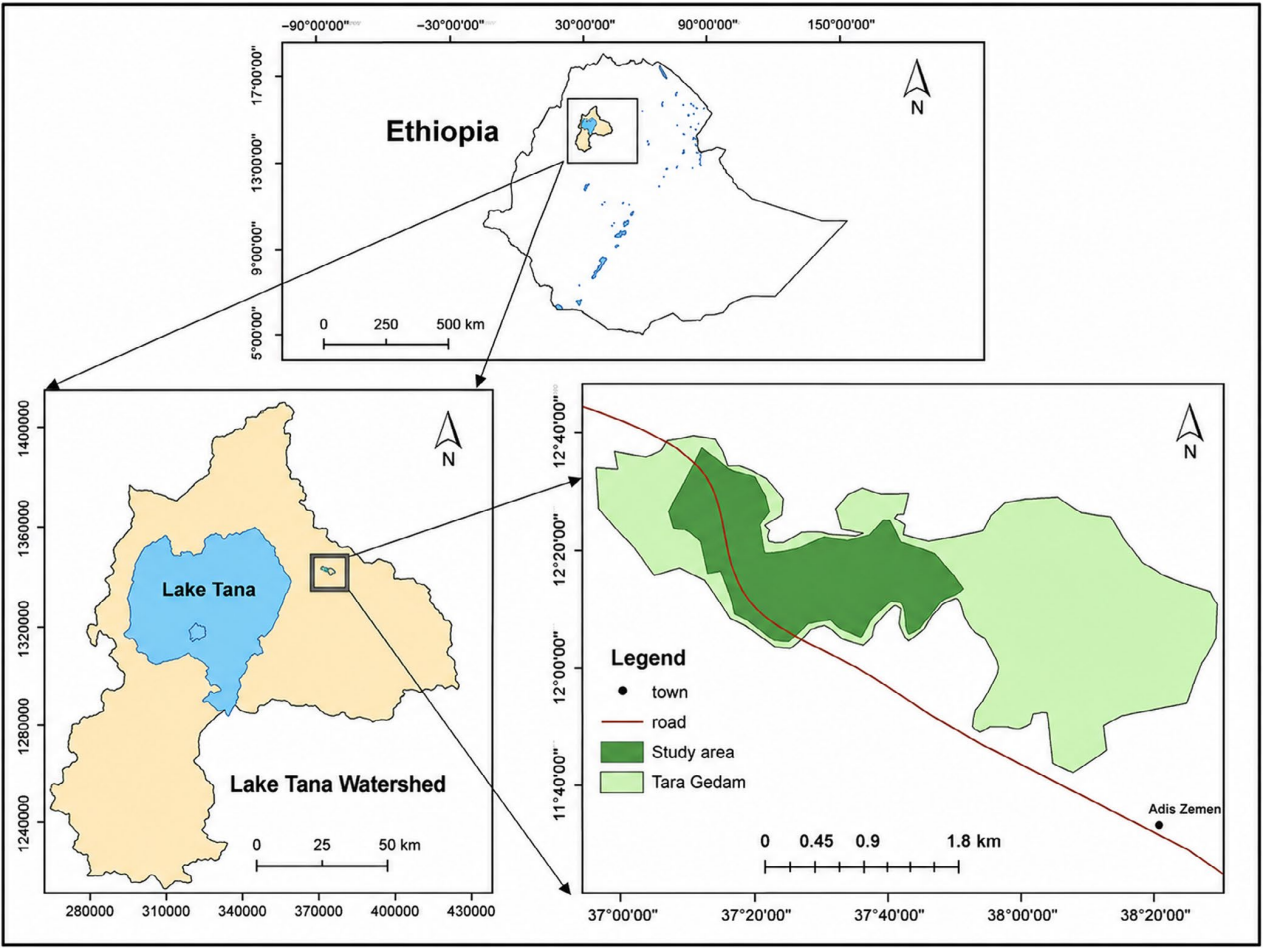
Map of Tara Gedam monastery church sacred forest.

The study was conducted in Tara Gedam sacred forest, in the Lake Tana watershed, located northeast of Lake Tana; 82 km north of Bahir Dar City. Geographically, it is located at 12°06°59° E, 12°07°25° N Latitude and 37°46°14° E, 37°47°02° E Longitude (Figure 1). It ranges from 2196 to 2457 m above sea level. The forest covers 815 ha (Kindu et al. 2022, Chapter 8).

Nestled in a moderate *Woina Dega* climate zone, Tara Gedam boasts a diverse ecosystem shaped by its unimodal rainfall and a mean annual temperature of 20.55°C. The landscape is a mosaic of natural forest, shrubland, and plantations, notably of *Cupressus lusitanica* introduced during the Derg regime (1974–1991). Its rich flora includes 41 documented woody species, with *Olea europaea* , *Allophylus abyssinicus*, and *Albizia schimperiana* forming the dominant canopy (Gedefaw and Soromessa 2014). Local reports and regional biodiversity assessments indicate the presence of large mammals such as leopards and striped hyenas, alongside numerous birds, insects, reptiles, and amphibians. Consequently, the forest is defined not only by its tree cover, where specimens exceed 5 m, but by the intricate web of life sustained by its unique climatic and vegetative conditions.

### 2.2 Field Methods

A preliminary survey was conducted during July 2019. The physical and vegetation features of the study area were assessed using ground survey. Then, the study area habitats were stratified into three classifications: *forest edge*, which includes the 300 m inside from the edge, *intermediate forest area* between 300 and 600 m, and the *interior forest* one is located next to the intermediate forest area found within 600 and 900 m. Since the forest is characterized by a steep slope and rugged terrain, with altitudes ranging from 2196 to 2457 m above sea level. The steep slope and disturbances such as settlement and agriculture around the forest periphery further shape habitat conditions. To capture gradients in avian community structure, the forest was stratified into three distance‐based zones. Although edge effects can extend beyond 300 m, this stratification balances ecological relevance with the forest's spatial constraints (≈800 ha) and logistical feasibility. Importantly, the terms “edge,” “intermediate,” and “interior” are applied in relative rather than absolute terms, reflecting the scale of this particular forest. Sampling plots (blocks) were distributed on the basis of the stratified random sampling technique. All sample sizes are equivalent across edge, intermediate, and interior forests.

Twenty sample blocks total, each covering 3.6 ha, were taken in a proportionate distribution throughout the three habitat stratifications. There were 10 point count stations indicated in each sampling block, which make a total of 200 point count observations made for each season. The 20 blocks were allocated for each cardinal direction (five each); however, the number of point stations allocated was: the “edge and intermediate” strata had 67 point stations each, while the interior had 66 depending on the terrain characteristics. To prevent counting the same member of a species more than once, each point count station was at least 300 m apart (Aynalem and Bekele 2009).

Because all three of the forest habitat classes include extensive vegetation cover (Gregory et al. 2004), the point count approach was used because it is thought to be more appropriate for sampling cryptic, shy, and skulking species in forest habitats. Every 3 days of the week, twice a day bird observations were conducted at each site. The majority of the bird species are active in the morning (6:30 a.m.—10:00 a.m.) and in the afternoon (4:00 p.m. and 6:30 p.m.) when the data were taken. Individual bird counts were taken at the point station when they were sighted. Data were collected between August 2019 and February 2020, taking into account both the wet (August–October) and dry seasons (December–February). Data collection was limited to diurnal species only. Bird traits such as plumage pattern, size, shape, color, songs, and calls were used to identify the species found in the research region (Aynalem and Bekele 2009). Bird identification and counting were done using direct observations and a pair of binoculars (10 × 40). A maximum of 30 m radius from a fixed point was applied for 10 min at the point count station. The location of each point was indicated, and the observed avian location was recorded using GPS. Throughout the study, each point count station was visited 2 times per season. Avian species were identified in the field using distinguishing characteristics and, in some cases, referring to field guides (Redman et al. 2009).

Bird guilds were classified following the hierarchical framework proposed by González‐Salazar et al. (2014), which organizes species according to three principal criteria: primary food type, foraging substrate, and activity period. Since all species recorded in this study were diurnal, the classification emphasized diet and foraging stratum as the key determinants of guild membership.

Data on vegetation and habitat structure were gathered at each bird survey station. A 20 m × 20 m quadrant (plot) was randomly placed on each bird data collection block to synchronize vegetation and bird data. Plots were divided into sixteen 5 m × 5 m subplots to improve measurement efficiency and reduce errors (Hurst and Allen 1993). The number of trees/shrubs in each quadrant, as well as their diameter at breast height, was measured. The diameters of trees and shrubs that are branched around the breast height were measured separately and averaged.

### 2.3 Data Analysis

#### 2.3.1 Bird Diversity and Abundance

The diversity indices were analyzed using PAST version 4.03 (Hammer et al. 2001). The Shannon–Wiener diversity index (H′) was calculated using the formula Weaver (1963); Spellerberg and Fedor (2003). These biodiversity indices were chosen to address the first and the second hypotheses.

(1) $$H^{\prime }=-\sum_{k=0}^{n}\left(\mathrm{pi}\;\text{lnpi}\right)$$

where *H*′ represents Shannon‐Wiener Index of Diversity, *pi* is the proportion of individuals of a given species to the total number of individuals, and ln indicates the natural logarithm.

Species evenness was assessed using Shannon's equitability index (*H'E*), Pielou (1966), calculated as follows:

(2) $$H^{\prime }E=\frac{H^{\prime }}{\text{Hmax}}$$

where *H max* = ln(*S*), and *S* is the number of bird species in the sampling plot.

We have also used Chao‐1 and iChao‐1, which both estimators are used to estimate species richness, particularly in ecological studies where not all species are observed (Chao 1984; Chao et al. 2016). The results were compared with the species richness of each habitat structure and season.

(3) $$\text{SChao}1=\text{Sobs}+\frac{F12}{2F2}$$

where: *Sobs* = observed bird richness, *F*1 = number of bird species seen only once (singletons), *F*2 = number of bird species observed exactly twice (doubletons).

The formula for iChao‐1 is similar to Chao‐1 but includes adjustments for the variance of the estimates:

(4) $$S^{i}=\text{Sobs}+\frac{n1\left(n1-1\right)}{2\left(n2+1\right)}+\frac{\left(n1\left(n1-1\right)\;\left(n1-2\right)\right)}{6\left(n2+1\right)\left(n2+2\right)}$$

The estimated number of church forest bird species against the number of individuals' samples was estimated. The individual rarefaction curve was extrapolated to show interior and edge effects on bird richness during both seasons. Each forest patch was not analyzed individually due to the similarity in evenness and richness; instead, the focus was on seasonal variations and the effects of edge and interior habitats on diversity and richness. Nevertheless, the influence of vegetation characteristics on bird species abundance and diversity was observed across the three forest patches.

All statistical analyses and data visualization were conducted using R software (R Core Team 2024), an open‐source programming environment widely used for statistical computing and graphical rendering. The ggplot2 package (Wickham 2016) was employed to generate all bar charts, as it implements the grammar of graphics framework, allowing for flexible and publication‐quality visualization of diversity indices across seasonal categories. Data restructuring and transformation, including the conversion of wide‐format tables into long‐format data frames suitable for faceted plotting, were performed using the tidyr package (Wickham et al. 2024). Finally, the gridExtra package (Auguie 2017) facilitated the arrangement of multiple individual plots into a single composite figure, enabling the clear side‐by‐side comparison of indices such as Taxa_S, Shannon_H, evenness, equitability, and species richness estimators across wet season, dry season, and total sampling periods. Together, these tools provided a robust and reproducible workflow for visualizing avian diversity patterns derived from field sampling data.

#### 2.3.2 Multivariate Analysis and Variance

To investigate the effects of habitat classes (forest interior, intermediate, and edge) from the perspective of vulnerability to disturbance level, distance from the edge, tree density, and tree DBH, we used general linear model (Multivariate Analysis of Covariance (MANCOVA) and tests of between subjects effects). In the MANCOVA, habitat class was a fixed factor whereas distance from the edge, tree density, and tree DBH were covariates. Before running this statistical test, assumptions were tested. The Box's Test of Equality of Covariance Matrices indicated a homogeneity of covariance matrices across groups (*M* = 11.33, *F* (6, 2622.761) = 1.543, *p* = 0.160).

The Levene's Test of Equality of Error Variances also supports the assumption that homogeneity of variance on the dependent variables is equal across groups: *F* (2, 17) = 2.34, *p* = 0.126 for bird species richness and *F* (2, 17) = 0.43, *p* = 0.958 for bird abundance.

Post hoc tests were conducted to test significant differences between habitat classes. The post hoc test considered the estimated marginal mean (adjusted mean) which is the mean of the dependent variables (bird species richness and bird abundance) for a particular level of the independent factor (habitat classes), after adjusting for the influence of the covariates (distance from the edge, tree density and tree DBH). The statistical tests were done at 0.05 significance level, but for the post hoc test we used the Bonferroni correction to adjust the significance level for pairwise comparison by dividing the significance level by the number of tests, that is, 0.05/3 = 0.016.

All statistical analyses were performed in SPSS version 26 for multivariate and ANOVA procedures, while diversity indices were calculated using PAST software (Hammer et al. 2001). Graphical visualizations derived from the diversity tables were produced in R software (R Core Team 2024), an open source environment widely used for statistical computing and rendering.

## 3 Results

### 3.1 Bird Diversity

**FIGURE 2:**
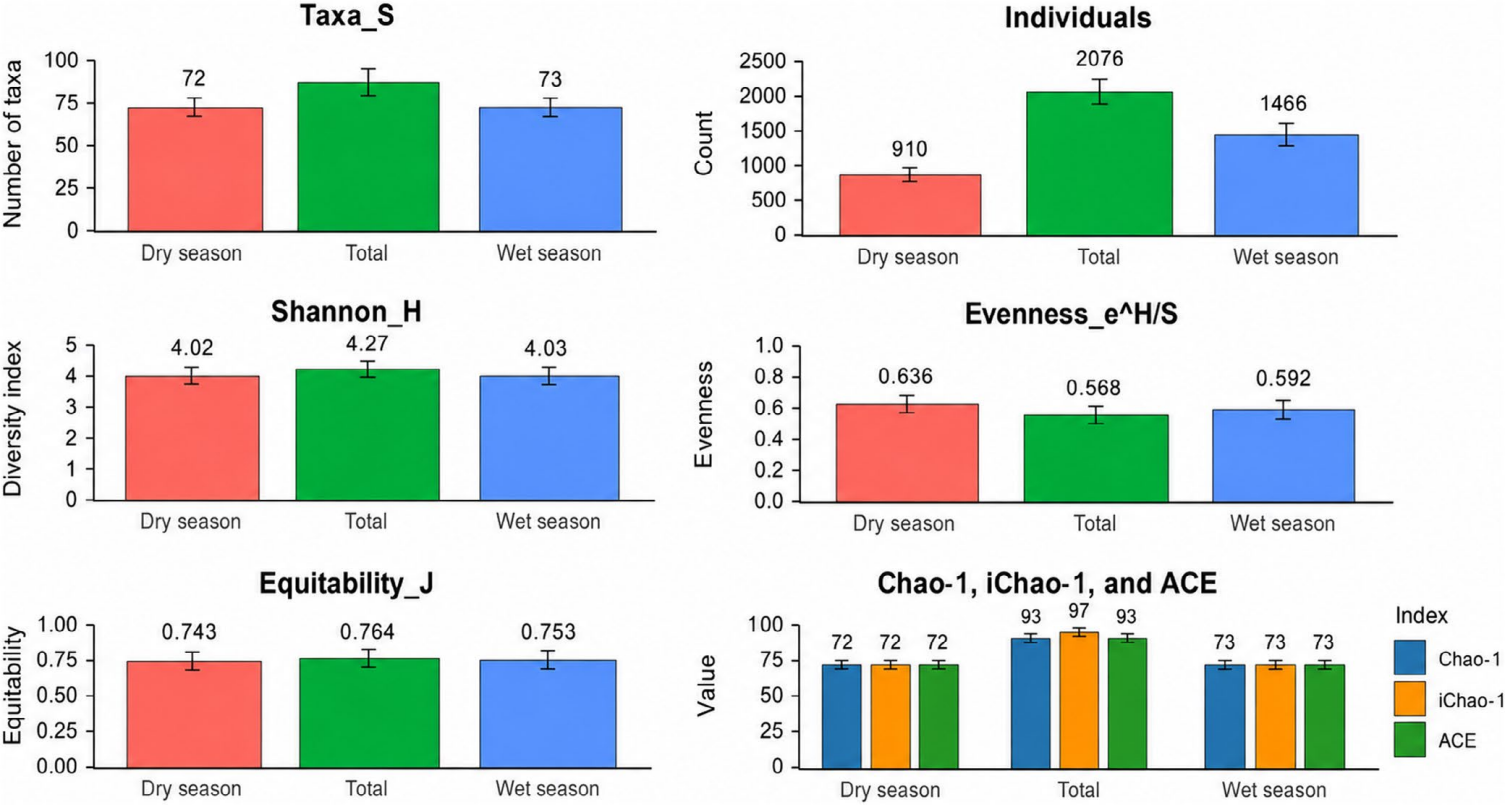
Bar charts showing avian diversity indices across wet season, dry season, and total sampling periods. (a) Taxa_S (number of taxa), (b) total individuals counted, (c) Shannon_H diversity index, (d) Evenness (*e*^*H*/*S*), (e) Equitability_J, and (f) grouped bar chart of Chao‐1, iChao‐1, and ACE species richness estimators. All values were derived from field sampling data. Note that Chao‐1, iChao‐1, and ACE produced identical values for each sampling period, reflecting the completeness of species detection.

The diversity analysis of bird species in the Tara Gedam Monastery church forest reveals a rich avian community, comprising 94 distinct species and a total of 2375 individual birds observed (Figure 2, Table S1). The Shannon Diversity Index (*H*) is 4.009, reflecting a significant level of diversity that accounts for both species abundance and evenness. With an evenness value of 0.586, the distribution of individuals among species is relatively balanced, although some species are more abundant than others. The equitability index (*J*) of 0.8824 demonstrates that the community is quite equitable, meaning that no single species significantly dominates the structure. Both the Chao‐1 and iChao‐1 estimates are 94, reinforcing the accuracy of the observed species richness and indicating that there are likely no significant numbers of unrecorded species. Finally, the ACE (Abundance‐based Coverage Estimator) value of 94 confirms that the species richness estimate is robust and reflective of the true diversity present.

During the wet season, the bird diversity analysis in the Tara Gedam church forest reveals a community consisting of 73 distinct species, with a total of 1466 individual birds observed. The Shannon Diversity Index (H) is calculated at 3.873, indicating a substantial level of diversity that reflects variations in species abundance and distribution. The evenness index (*e*^*H*/*S*) is measured at 0.6587, suggesting a moderate distribution of individuals among the species, where some species are more prevalent than others but overall diversity is maintained. The equitability index (J) is notably high at 0.9027, indicating that the species are relatively evenly represented within the community, contributing to a balanced ecological structure. Both the Chao‐1 and iChao‐1 estimates stand at 73, confirming the observed species richness and suggesting that the number of unrecorded species is minimal. The ACE (Abundance‐based Coverage Estimator) value of 73 further supports the robustness of the species richness estimate. Overall, these results indicate that the bird community in the Tara Gedam church forest during the wet season is diverse and well‐distributed, characterized by a healthy mix of species and a balanced ecological environment.

During the dry season, the bird diversity analysis in the Tara Gedam church forest shows a total of 72 distinct species, with 910 individual birds recorded (Figure 2). The Shannon Diversity Index (H) is 3.905, indicating a strong level of diversity within the community, reflecting both the abundance and variety of species present. The evenness index (*e*^*H*/*S*) is measured at 0.6896, suggesting a relatively balanced distribution of individuals among the species, with some species being more common while still maintaining overall diversity. The equitability index (*J*) is high at 0.9131, demonstrating that the species are evenly represented, which contributes to a well‐structured ecological community. Both the Chao‐1 and iChao‐1 estimates are 72, confirming the observed species richness and indicating that the likelihood of unrecorded species is low. The ACE (Abundance‐based Coverage Estimator) value of 72 further supports the reliability of the species richness estimate.

### 3.2 Bird Species Richness and Abundance Associated With Edge Effect and Vegetation Characteristics

The multivariate analysis of covariance shows that the interaction of habitat class, distance from the edge, tree density and tree DBH had a statistically significant overall effect on the bird species richness and bird abundance (Roy's largest root = 1.731, *F* (3, 11) = 6.346, *p* = 0.009, *η* ^2^ = 0.634). The significant interaction result indicates that the effect of habitat class (forest interior, intermediate and edge) on the changes in bird species richness and abundance depends on the values of distance from the edge, tree density and tree DBH.

Further univariate test for the interaction effect of these predictor variables on the abundance of birds was also statistically significant (*F* = 6.346, *p* = 0.009, *η* ^2^ = 0.634). The effect size of the interaction of habitat classes, distance from the edge, tree density, and tree DBH on the bird abundance was stronger than the main effect of each factor/covariate (Table 1). However, the interaction effect on the bird species richness was not statistically significant (*F* = 2.359, *p* = 0.128, *η* ^2^ = 0.392). Therefore, the main effect of factors on the bird abundance was interpreted separately.

**TABLE 1:** Results of multivariate analysis (MANOVA) using Roy's largest root statistic for the effects of habitat class, distance from edge, tree density, and tree DBH on the dependent variable (richness and abundance) set.

Multivariate tests^a^							
Effect		Value	*F*	Hypothesis df	Error df	Sig.	Partial eta squared
Intercept	Roy's largest root	0.280	1.399^b^	2.000	10.000	0.291	0.219
Habitat Class	Roy's largest root	1.787	9.831^c^	2.000	11.000	0.004	0.641
Distance from the edge	Roy's largest root	1.176	5.878^b^	2.000	10.000	0.021	0.540
Tree density	Roy's largest root	0.409	2.044^b^	2.000	10.000	0.180	0.290
Tree DBH	Roy's largest root	0.917	4.585^b^	2.000	10.000	0.039	0.478
HClass * distancem * AvDensity400m2 * AvDBHcm	Roy's largest root	1.731	6.346^c^	3.000	11.000	0.009	0.634

^a^ Design: Intercept + HClass + distancem + AvDensity400m^2^ + AvDBHcm + HClass * distancem * AvDensity400m^2^ * AvDBHcm.

^b^ Exact statistic.

^c^ The statistic is an upper bound on F that yields a lower bound on the significance level.

The change on bird species richness was statistically significantly influenced by distance from the edge (*F* = 7.934, *p* = 0.007, *η* ^2^ = 0.591), whereas the effect of habitat class (*F* = 0.432, *p* = 0.66, *η* ^2^ = 0.073), tree density (*F* = 0.026, *p* = 0.87, *η* ^2^ = 0.002), and tree DBH (*F* = 4.481, *p* = 0.058, *η* ^2^ = 0.289) on bird species richness was not significant. The effect of habitat class (*F* = 7.934, *p* = 0.007, *η* ^2^ = 0.591), distance from the edge (*F* = 5.775, *p* = 0.035, *η* ^2^ = 0.344), and tree DBH (*F* = 8.144, *p* = 0.016, *η* ^2^ = 0.425) on bird abundance was significant, while tree density (*F* = 3.965, *p* = 0.072, *η* ^2^ = 0.265) did not have a significant effect on bird abundance (Table 2).

**TABLE 2:** Univariate results (ANOVA) from the between‐subjects effects tests for bird species richness and abundance.

Tests of between‐subjects effects							
Source	Dependent variable	Type III sum of squares	df	Mean square	*F*	Sig.	Partial eta squared
Corrected Model	Bird species richness	1021.571^a^	8	127.696	137.326	0.000	0.990
	Bird abundance	72,718.974^b^	8	9089.872	188.010	0.000	0.993
Intercept	Bird species richness	0.639	1	0.639	0.687	0.425	0.059
	Bird abundance	141.893	1	141.893	2.935	0.115	0.211
HClass	Bird species richness	0.803	2	0.401	0.432	0.660	0.073
	Bird abundance	767.165	2	383.582	7.934	0.007	0.591
Distance m	Bird species richness	9.685	1	9.685	10.415	0.008	0.486
	Bird abundance	279.194	1	279.194	5.775	0.035	0.344
AvDensity400m^2^	Bird species richness	0.024	1	0.024	0.026	0.875	0.002
	Bird abundance	191.676	1	191.676	3.965	0.072	0.265
AvDBHcm	Bird species richness	4.167	1	4.167	4.481	0.058	0.289
	Bird abundance	393.729	1	393.729	8.144	0.016	0.425
HClass * distance m * AvDensity400m^2^ * AvDBHcm	Bird species richness	6.581	3	2.194	2.359	0.128	0.392
	Bird abundance	920.420	3	306.807	6.346	0.009	0.634
Error	Bird species richness	10.229	11	0.930			
	Bird abundance	531.826	11	48.348			
Total	Bird species richness	10,624.000	20				
	Bird abundance	339,594.000	20				
Corrected total	Bird species richness	1031.800	19				
	Bird abundance	73,250.800	19				

^a^ *R* ^2^ = 0.990 (Adjusted *R* ^2^ = 0.983).

^b^ *R* ^2^ = 0.993 (Adjusted *R* ^2^ = 0.987).

The bird species show a significant difference between edge versus interior (adjusted mean difference = 48.614, *p* = 0.005), while controlling for the influence of distance from the edge, tree density, and tree DBH. However, the difference between edge versus intermediate (adjusted mean difference = 41.650, *p* = 0.037) and intermediate versus interior (adjusted mean difference = 6.964, *p* = 0.196) was not statistically significant. In addition, the difference in bird abundance between edge versus intermediate (adjusted mean difference = 349.745, *p* = 0.015) and edge versus interior (adjusted mean difference = 328.568, *p* = 0.007) was significant, whereas the difference between intermediate versus interior (adjusted mean difference = −21.177, *p* = 1.00) was not statistically significant (Table 3).

**TABLE 3:** Pairwise comparisons of bird species richness and abundance among habitat classes (edge, intermediate, interior).

Pairwise comparisons							
Dependent variable	(*I*) Habitat class	(*J*) Habitat class	Mean difference (*I*−*J*)	SE	Sig.^a^	95% Confidence interval for difference^a^	
						Lower bound	Upper bound
Bird species richness	Edge	Intermediate	41.650*	13.935	0.037	2.354	80.947
		Interior	48.614*	11.618	0.005	15.852	81.376
	Intermediate	Edge	−41.650*	13.935	0.037	−80.947	−2.354
		Interior	6.964	3.401	0.196	−2.628	16.556
	Interior	Edge	−48.614*	11.618	0.005	−81.376	−15.852
		Intermediate	−6.964	3.401	0.196	−16.556	2.628
Bird abundance	Edge	Intermediate	349.745*	100.479	0.015	66.392	633.098
		Interior	328.568*	83.771	0.007	92.331	564.805
	Intermediate	Edge	−349.745*	100.479	0.015	−633.098	−66.392
		Interior	−21.177	24.526	1.000	−90.342	47.988
	Interior	Edge	−328.568*	83.771	0.007	−564.805	−92.331
		Intermediate	21.177	24.526	1.000	−47.988	90.342

*Note:* Based on estimated marginal means.

^a^ Adjustment for multiple comparisons: Bonferroni.

* The mean difference is significant at the 0.05 level.

**FIGURE 3:**
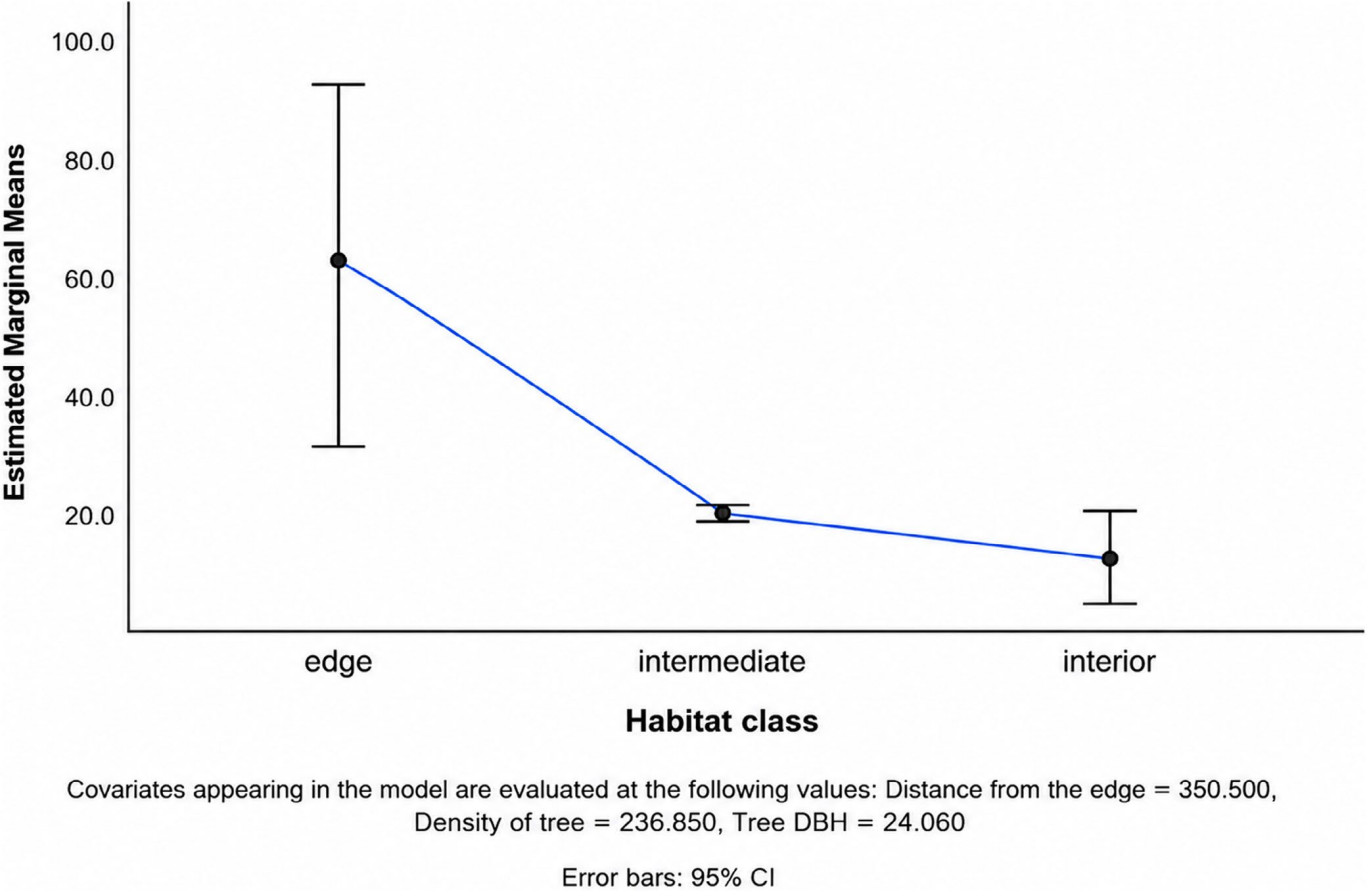
Estimated marginal means of bird species richness (±95% CI) for three habitat classes (edge, intermediate, interior). Covariates (distance from the edge, tree density, tree DBH) are held constant at their mean values.

The estimated marginal mean indicates both the species richness and abundance were higher in the forest edge than the intermediate and interior (Figure 3).

### 3.3 Foraging Guilds

The bird community in the Tara Gedam forest displays a diverse array of foraging guilds, highlighting the ecological complexity of the area. Among the observed species, omnivores are the most prevalent, with 38 individuals. Following closely are the insectivores, comprising 25 species. Granivores, or seed‐eating birds, number 18. The carnivorous birds number 11, while frugivores with just 2 species.

The foraging strata of the bird community in the Tara Gedam church forest reveal a diverse and stratified use of habitat that reflects the ecological dynamics of the area. Arboreal foragers dominate the community, with 39 species utilizing the canopy and branches of trees to find food. Ground foragers are also significant, with 41 species observed foraging on the forest floor. Aerial foragers, comprising 11 species, utilize the air space, likely preying on flying insects and participating in pollination. Lastly, the presence of 3 species that forage near water indicates that aquatic resources are also utilized, although to a lesser extent.

## 4 Discussion

The avian community within the Tara Gedam Monastery church forest exhibits remarkably high and stable biodiversity based on the results found. The total number of species recorded, yielding a high Shannon Diversity Index, underscores the presence of a rich and ecologically complex bird assemblage within this forest fragment. High equitability indices, coupled with the close agreement between observed species richness and estimator values (Chao‐1, ACE), indicate that the community is not only species‐rich but also well‐structured, with no single species dominating (Zelelew et al. 2025). These findings align with the globally recognized role of Ethiopian church forests as critical refugia for biodiversity within fragmented agricultural landscapes (Aerts et al. 2008). More specifically, they support our initial hypothesis that the Tara Gedam forest, as an isolated sacred grove, would sustain a diverse avian assemblage characterized by high species richness and an even distribution of individuals (Kumar and Koli 2024, 2025; Areaya et al. 2013).

The most striking seasonal pattern was a 61% higher total bird abundance in the wet season compared to the dry season, a shift attributable to annual cycles of reproduction and resource availability. The wet season coincides with peaks in insect abundance (Tanaka and Tanaka 1982; Kumari and Priya 2022), fruit production (Levey 1988), and nesting activity (Hurlbert and Haskell 2003), which attract and support a larger number of individuals, including migrants and breeding residents.

Despite this substantial disparity in abundance, species richness and Shannon diversity remained remarkably stable between seasons, a pattern of diversity consistency also observed in other tropical systems (Nieto et al. 2026). Interestingly, evenness was slightly higher during the dry season, suggesting an ecological adjustment in community structure. As overall resource availability declines, the bird community may become more evenly distributed across the forest's stable microhabitats, potentially reflecting a shift toward generalist or drought‐tolerant species.

This seasonal stability in diversity does not support our second hypothesis, which predicted that avian community structure would vary between wet and dry seasons due to seasonal pulse of resource availability (Levey 1988). Instead, the findings indicate that the protected status of the church forest plays a central role in maintaining ecological function by providing a perennial sanctuary for birds (Habel et al. 2025).

However, our recent study on the “Effect of seasonal variations and vegetation on bird composition and foraging guilds in selected church forests of the southwestern Lake Tana area” revealed that season did impact avian community structure (Zelelew et al. 2025). In that study, the three selected church forests were isolated, patchy, and smaller in size (Zelelew et al. 2025), in contrast to the Taragedam Monastery Church forest, which is intact and extensive (> 800 ha). The larger forest cover and intact nature of the Taragedam Monastery Church forest likely contribute to the stability of avian community structure across seasons.

The contrasting findings between fragmented, isolated forests and intact continuous forests are well supported by the literature. Habitat fragmentation and isolation fundamentally alter how avian communities respond to seasonal changes through several mechanisms. First, isolation distance imposes dispersal limitations that disproportionately affect sedentary forest specialists. In a study of fragmented forest systems in South Africa, Ehlers Smith et al. (2018) demonstrated that sedentary forest specialists were unable to disperse across isolation distances exceeding 500 m, whereas migratory forest specialists exhibited greater resilience to isolation effects during the non‐breeding season. This suggests that in fragmented landscapes, seasonal turnover may be exacerbated by the inability of sensitive species to track resources across the matrix. Second, the effects of fragmentation on community stability manifest differently across seasons. Research by Liu et al. (2025) in urban green spaces revealed that habitat fragmentation increased community stability indirectly through functional diversity in both breeding and winter seasons, yet decreased stability through population asynchrony specifically during the breeding season. This indicates that fragmented habitats may destabilize breeding bird populations while paradoxically providing refugia during non‐breeding periods. The authors concluded that “urban green space planning strategies should be seasonally adaptive,” with connecting fragmented habitats being essential in the breeding season to support functional diversity and stability (Liu et al. 2025). Third, patch area reduction consistently leads to declines in forest bird abundance regardless of season. Verga et al. (2020) found that population abundance of the overall avian community and of forest specialist species declined with patch area reduction independently of season, with reductions exceeding 65% for some forest species. Importantly, they documented that the negative effects of forest fragmentation were most pronounced during the breeding season, when resource demands are highest (Verga et al. 2020). Fourth, habitat amount in the surrounding landscape serves as a positive driver of avian diversity across seasons. Multiple studies have confirmed that the quantity of surrounding habitat mitigates isolation effects, with higher habitat amounts supporting greater taxonomic and functional diversity (Howe 1984; Leveau et al. 2021). In contrast, small isolated patches exhibit more predictable but less diverse assemblages over time, as transients and generalists replace forest specialists (Howe 1984).

The intact nature and extensive size (> 800 ha) of the Taragedam Monastery Church forest thus appear to buffer against the seasonal instability observed in smaller, fragmented church forests. By maintaining connectivity, providing continuous habitat structure, and supporting both resident and habitat‐sensitive species throughout the annual cycle, large protected forests serve as irreplaceable elements for avian conservation (Leveau et al. 2021). This aligns with broader evidence that protected areas maintaining forest integrity can stabilize bird communities across seasonal gradients, whereas fragmented habitats amplify seasonal variation and promote community homogenization (Ehlers Smith et al. 2018; Liu et al. 2025). Thus, the protected status of the Taragedam Monastery Church forest is central to maintaining ecological function by offering a perennial sanctuary that mitigates the seasonal resource pulses driving community change in more fragmented systems (Habel et al. 2025).

Our analysis reveals that avian responses are governed by complex interactions between habitat structure and edge effects, not by simple gradients. The most compelling result is the significant four‐way interaction (Habitat Class × Distance × Tree Density × Tree DBH) on the avian community (MANOVA, *η* ^2^ = 0.634). This large effect size indicates that the influence of forest position (edge vs. interior) is critically modified by local vegetation structure. For instance, an edge with low tree density and small DBH may attract open‐area species, while a dense, mature edge may not. This underscores the insufficiency of simple “edge vs. interior” models and highlights that the ecological quality of the edge is paramount (Laurance et al. 2007; Murcia 1995). Consequently, these results provide strong support for our third hypothesis, which posited that vegetation structure and edge proximity interact to shape avian community metrics.

Consistent with our hypothesis, the results revealed a significant negative correlation between distance from the core forest habitat and avian community metrics, supporting the ecological principle of distance decay. The multivariate analysis demonstrated that “distance from the edge” was a significant predictor of both species richness (*F* = 7.934, *p* = 0.007) and bird abundance (*F* = 5.775, *p* = 0.035). Specifically, pairwise comparisons showed that species richness was significantly higher in the edge habitat compared to the interior (adjusted mean difference = 48.614, *p* = 0.005), with a similar trend observed for bird abundance (adjusted mean difference = 328.568, *p* = 0.007). Contrary to classic edge effect theory predicting biotic depletion, both species richness and abundance were significantly higher at the forest edge. This finding further supports our third hypothesis by demonstrating that the edge‐interior transition, mediated by vegetation structure, significantly influences avian community metrics. This “ecotonal effect” arises because the abrupt transition creates a unique habitat that attracts species from both adjacent ecosystems (Kark and Van Rensburg 2006). The edge's enhanced vertical complexity, where increased light penetration promotes denser understory growth (Harper et al. 2005), supports a greater diversity of foraging niches, attracting both forest‐interior and matrix species (Ries and Sisk 2004).

The analysis of foraging guilds and strata reveals the mechanistic basis for the high biodiversity: trophic and spatial niche partitioning. The community exhibits a balanced, insect‐driven trophic structure. The prevalence of insectivores (25 species) and omnivores (38 species) indicates a food web heavily based on abundant arthropod prey, a hallmark of structurally complex forests (Sekercioglu 2012). The low number of specialist frugivores (2 species) suggests seed dispersal is likely provided by resilient omnivores like bulbuls and starlings, a functional shift common in fragmented systems (Corlett 2017; Mokotjomela et al. 2013). In contrast, the significant granivore guild (18 species) points to important grass and seed resources from the forest floor and edge (Fisher and Davis 2010). The presence of carnivores, including raptors (11 species), indicates a relatively complete trophic structure, providing essential top‐down regulation and serving as indicators of ecosystem health (Terborgh et al. 2001).

Spatially, the near‐equal representation of arboreal (39 species) and ground (41 species) foragers is a direct testament to the forest's vertical structural heterogeneity. This stratification is a classic mechanism for reducing interspecific competition (MacArthur 1958). A multi‐layered canopy (supported by large trees) niches for arboreal foragers, while a developed understory (influenced by tree density and edge proximity) supports ground foragers. This structured use of space allows for the “niche packing” characteristic of stable, mature ecosystems.

The guild and strata analysis moves beyond species lists to explain coexistence. The results depict a functionally diverse and compartmentalized community where birds partition resources by food type and foraging space. This functional diversity enhances ecosystem resilience (Sekercioglu 2006). Crucially, the reliance of specific guilds on specific structures means conservation must preserve structural complexity in its entirety. The loss of large trees would disproportionately affect arboreal guilds (Lindenmayer and Laurance 2017), while understory removal would devastate ground foragers. Such simplification acts as an extinction filter for specialists (Betts et al. 2019).

Overall, the Tara Gedam Monastery forest is not merely a species refuge but a fully functioning, stratified ecosystem. Its high taxonomic diversity is underpinned by significant functional diversity, maintained through niche partitioning supported by complex habitat structure. This ecological integrity is a direct product of long‐term, faith‐based protection.

## 5 Conclusion

This study demonstrates that the Tara Gedam Monastery church forest supports a rich, stable, and functionally complex avian community, affirming its critical role as a biodiversity refuge within a fragmented landscape. The high taxonomic diversity (94 species) and stable species richness across seasons are direct outcomes of the forest's long‐term protected status, which maintains perennial resources and a buffered microclimate.

The avian community is structured through a dual mechanism of trophic and spatial niche partitioning. An insect‐driven trophic pyramid, supported by abundant insectivores and omnivores, forms the base of a food web that extends to specialized granivores and a complete carnivore guild. Spatial stratification is equally vital, with nearly equal numbers of arboreal and ground foragers coexisting within the forest's vertically complex structure. This partitioning minimizes competition and is a hallmark of a mature, resilient ecosystem.

Crucially, bird distribution and abundance are governed not by simple edge versus interior gradients, but by complex interactions between habitat structure and edge context. The significant influence of tree DBH, density, and distance from the edge, culminating in a strong four‐way interaction, reveals that the forest edge functions as a productive ecotone rather than a degraded margin. Enhanced structural heterogeneity at the edge creates diverse foraging niches, attracting species from both the forest interior and the surrounding matrix.

Therefore, the conservation value of Tara Gedam extends beyond its species list. It is a fully functioning, architecturally complex ecosystem where ecological processes such as predation, seed dispersal, and competition unfold with minimal anthropogenic disruption. Effective conservation must focus on preserving this structural complexity in its entirety, from large canopy trees to dense understory, and recognize the ecological value of structured forest edges. The enduring integrity of this forest underscores that faith‐based protection can sustain ecological resilience, offering a vital model for conserving biodiversity in human‐modified landscapes worldwide.

## Author Contributions

**Shimelis Aynalem Zelelew:** conceptualization (equal), data curation (equal), formal analysis (lead), funding acquisition (supporting), investigation (supporting), methodology (lead), project administration (equal), resources (supporting), software (lead), supervision (lead), validation (lead), visualization (lead), writing – original draft (lead), writing – review and editing (lead). **Melkeneh Wondie:** conceptualization (supporting), data curation (lead), formal analysis (supporting), investigation (lead), methodology (equal), resources (supporting), visualization (supporting), writing – original draft (supporting), writing – review and editing (supporting). **Mezgebu Ashagire:** conceptualization (equal), data curation (equal), formal analysis (equal), funding acquisition (lead), investigation (supporting), methodology (supporting), project administration (supporting), resources (supporting), software (equal), supervision (supporting), validation (supporting), visualization (equal), writing – original draft (supporting), writing – review and editing (supporting).

## Conflicts of Interest

The authors declare no conflicts of interest.

## Supporting information


**Table S1:** Bird lists and foraging guilds based on diet and foraging strata, Tara Gedam forest.

## Data Availability

The data supporting the results of this study are available as Supporting Information in the online version of this article. Raw data files (e.g., bird abundance matrices and habitat variables) have been provided as Supporting Information.
