# Supplementary material for: Bird Diversity and Habitat Associations in the Tara Gedam Monastery Church Forest: A Seasonally Informed Study in Northwestern Ethiopia
Source: Ecol Evol. 2026 Jun 9;16(6):e73789. doi: 10.1002/ece3.73789 (PMC13249529; doi:10.1002/ece3.73789)
Supplement: Supplementary file 1 — Table S1: Bird lists and foraging guilds based on diet and foraging strata, Tara Gedam forest. [file ECE3-16-e73789-s001.docx]

Table S1. Bird lists and foraging guilds based on diet and foraging strata, Tara Gedam forest

| Common name | Scientific name | Diet | Foraging stratum |
| --- | --- | --- | --- |
| Abyssinian Catbird | *Parophasma galinieri* | Omnivores | Arboreal |
| Abyssinian Ground-Hornbill | *Geokichla piaggiae* | Omnivores | Ground feeders |
| Abyssinian oriole | *Oriolus monacha* | Insectivores | Arboreal |
| Abyssinian Owl | *Asio abyssinicus* | Carnivores | Arboreal |
| Abyssinian Slaty-Flycatcher | *Melaenornis chocolatinus* | Insectivores | Arboreal |
| Abyssinian White-eye | *zosterops abyssinicus* | Omnivores | Arboreal |
| Abyssinian Woodpecker | *Dendropicos abyssinicus* | Omnivores | Arboreal |
| African Citril | *Crithagra citrinelloides* | Granivores | Arboreal |
| African Gray Hornbill | *Tockus nasutus* | Omnivores | Arboreal |
| African Harrier-Hawk | *Polyboroides typus* | Carnivores | Arboreal |
| African Paradise-Flycatcher | *Terpsiphone viridis* | Insectivores | Arboreal |
| African Thrush | *Turdus pelios* | Omnivores | Ground feeders |
| Augur Buzzard | *Buteo augur* | Carnivores | Aerial |
| Baglafecht Weaver | *Ploceus baglafecht* | Omnivores | Arboreal |
| Banded Barbet | *Lybius undatus* | Omnivores | Arboreal |
| Black-billed barbet | *Lybius guifsobalito* | Omnivores | Arboreal |
| Black-breasted Snake-Eagle | *Circaetus pectoralis* | carnivores | Areal |
| Black-headed Batis | *Batis minor* | Insectivores | Arboreal |
| Black-winged Lovebird | *Agapornis taranta* | Granivores | Arboreal |
| Black-winged red Bishop | [*Euplectes hordeaceus*](https://birdsoftheworld.org/bow/species/blwbis1/cur/introduction#etymologyDefs) | Omnivores | Ground feeders |
| Blue-breasted Bee-eater | *Merops variegatus* | Insectivores | Aerial |
| Brown Woodland-Warbler | *Phylloscopus umbrovirens* | Insectivores | Arboreal |
| Brown-rumped Seadeater | *Crithagra tristriata* | Granivores | Arboreal |
| Cape Rook | *Corvus capensis* | Omnivores | Ground feeders |
| Cardinal woodpecker | *Dendropicos fuscescens* | Omnivores | Arboreal |
| Cattle Egret | *Ardea ibis* | Carnivores | Ground feeders |
| Clappertons francolin | *Pternistis clappertoni* | Granivores | Ground feeders |
| Common Bulbul | *Pycnonotus barbatus* | Omnivores | Arboreal |
| Common cockoo | *Cuculus canorus* | Insectivores | Aerial |
| Common Fiscal | *Lanius collaris* | Omnivores | Ground feeders |
| Common Redstart | *Phoenicurus phoenicurus* | Omnivores | Ground feeders |
| Common swift | *Apus apus* | Insectivores | Aerial |
| Common Waxbill | *Estrilda astrild* | Omnivores | Ground feeders |
| Dark Chanting-Goshawk | *Melierax metabates* | Carnivores | Aerial |
| Eurasian hoopoe | *Upupa epops* | Insectivores | Ground feeders |
| Exclamatory Paradise-Whydah | *Vidua interjecta* | Granivores | Ground feeders |
| Fan-tailed Raven | *Corvus rhipidurus* | Omnivores | Ground feeders |
| Greater Blue-eared Starling | *Lamprotornis chalybaeus* | Omnivores | Arboreal |
| Hadada Ibis | *Bostrychia hagedash* | Insectivores | Water |
| Helmeted Guineafowl | *Numida meleagris* | Omnivores | Ground feeders |
| Hemprich's Hornbill | *Lophoceros hemprichii* | Omnivores | Arboreal |
| Hooded Vulture | *Necrosyrtes monachus* | Carnivores | Ground feeders |
| Laughing Dove | *Streptopelia senegalensis* | Omnivores | Ground feeders |
| Lemon Dove | *Columba larvata* | Omnivores | Arboreal |
| Lesser Blue-eared Starling | *Lamprotornis chloropterus* | Omnivores | Ground feeders |
| Little Bee-eater | *Merops pusillus* | Insectivores | Aerial |
| Little Weaver | *Ploceus luteolus* | Omnivores | Arboreal |
| Mountain Thrush | *Turdus plebejus* | Omnivores | Ground feeders |
| Mountain Wagtail | *Motacilla clara* | Insectivores | Ground feeders |
| Namaqua Dove | *Oena capensis* | Granivores | Ground feeders |
| Narina Trogon | *Apaloderma narina* | Insectivores | Arboreal |
| Northern Red Bishop | *Euplectes franciscanus* | Granivores | Ground feeders |
| Nubian Woodpecker | *Campethera nubica* | Insectivores | Arboreal |
| Nyanza Swift | *Apus niansae* | Insectivores | Aerial |
| Pale Flycatcher | *Muscicapa pallida* | Insectivores | Arboreal |
| Pied Crow | *Corvus albus* | Omnivores | Ground feeders |
| Pied Wheatear | *Oenanthe pleschanka* | Insectivores | Ground feeders |
| Pin-tailed Whydah | *Vidua macroura* | Granivores | Ground feeders |
| Red-backed shrike | Lanius collurio | Omnivores | Arboreal |
| Red-billed Firefinch | *Lagonosticta senegala* | Granivores | Ground feeders |
| Red-breasted Wheatear | [Oenanthe bottae](https://birdsoftheworld.org/bow/species/rebwhe2/cur/introduction#etymologyDefs) | Omnivores | Ground feeders |
| Red-cheeked Cordonbleu | *Uraeginthus bengalus* | Granivores | Ground feeders |
| Red-collared Widowbird | *Euplectes ardens* | Omnivores | Ground feeders |
| Red-eyed dove | *Streptopelia semitorquata* | Granivores | Ground feeders |
| Red-winged Starling | *Onychognathus morio* | Omnivores | Arboreal |
| Rock Martin | *Ptyonoprogne fuligula* | Insectivores | Aerial |
| Rüppell's Robin-Chat | *Cossypha semirufa* | Insectivores | Ground feeders |
| Sacred Ibis | *Threskiornis aethiopicus* | Omnivores | Water |
| Sand Martin | *Riparia riparia* | Insectivores | Aerial |
| Scarlet-chested Sunbird | *Chalcomitra senegalensis* | Omnivores | Arboreal |
| Shikra | *Accipiter badius* | Carnivores | Arboreal |
| Speckled Mousebird | *Colius striatus* | Frugivores | Arboreal |
| Speckled Pigeon | *Columba guinea* | Granivores | Ground feeders |
| Spectacled Weaver | *Ploceus ocularis* | Insectivores | Arboreal |
| Streaky Seedeater | *Serinus striolatus* | Granivores | Arboreal |
| Tacazze Sunbird | *Nectarinia tacazze* | Omnivores | Arboreal |
| Tawny Eagle | *Aquila rapax* | Carnivores | Ground feeders |
| Thick-billed Raven | *Corvus crassirostris* | Omnivores | Ground feeders |
| Tree Pipit | *Anthus trivialis* | Insectivores | Ground feeders |
| Variable Sunbird | *Cinnyris venustus* | Omnivores | Arboreal |
| Village Indigobird | *Vidua chalybeata* | Granivores | Ground feeders |
| Village Weaver | *Ploceus cucullatus* | Omnivores | Arboreal |
| Wattled ibis | *Bostrychia carunculata* | Insectivores | Water |
| White- throated seedeater | *Sporophila albogularis* | Granivore | Ground feeder |
| White-backed Vulture | *Gyps africanus* | Carnivore | Ground feeder |
| White-cheeked Turaco | *Menelikorins leucotis* | Frugivores | Arboreal |
| White-winged Cliff-Chat | *Thamnolaea semirufa* | Insectivores | Ground feeders |
| Willow Warbler | *Phylloscopus trochilus* | Omnivores | Arboreal |
| Yellow wagtail | *Motacilla flava* | Insectivore | Ground feeder |
| Yellow-billed Kite | *Milvus migrans aegyptius* | Carnivores | Aerial |
| Yellow-billed Waxbill | *Coccopygia quartinia* | Granivores | Ground feeders |
| Yellow-crowned Bishop | *Euplectes afer* | Omnivores | Ground feeders |
| Yellow-fronted Canary | *Serinus mozambicus* | Granivores | Arboreal |
| Yellow-mantled Widowbird | *Euplectes macrourus* | Granivores | Ground feeders |
